# Assessing bilateral ankle proprioceptive acuity in stroke survivors: An exploratory study

**DOI:** 10.3389/fneur.2022.929310

**Published:** 2022-08-11

**Authors:** Li Pan, Dongyan Xu, Weining Wang, Jifeng Rong, Jinyao Xu, Amanda Ferland, Roger Adams, Jia Han, Yulian Zhu

**Affiliations:** ^1^Department of Rehabilitation Medicine, Huashan Hospital Fudan University, Shanghai, China; ^2^School of Kinesiology, Shanghai University of Sport, Shanghai, China; ^3^The First Rehabilitation Hospital of Shanghai, Shanghai, China; ^4^Research Institute for Sport and Exercise, University of Canberra, Canberra, NSW, Australia; ^5^USC Division of Biokinesiology and Physical Therapy, Orthopedic Physical Therapy Residency, and Spine Rehabilitation Fellowship, The First Rehabilitation Hospital of Shanghai, Shanghai, China; ^6^College of Rehabilitation Sciences, Shanghai University of Medicine and Health Sciences, Shanghai, China; ^7^Faculty of Health, Arts and Design, Swinburne University of Technology, Melbourne, VIC, Australia

**Keywords:** stroke, proprioception, ankle, motor control, mobility, bilateral

## Abstract

**Background:**

Bilateral proprioception deficits were reported in stroke survivors. However, whether bilateral proprioception deficits exist in the ankle joint after stroke was unclear. Ankle proprioception is a significant predictor of balance dysfunction after stroke, and previous studies to date are lacking appropriate evaluation methods.

**Objectives:**

We want to determine whether the active movement extent discrimination apparatus (AMEDA) is a reliable tool for assessing ankle proprioceptive acuity in stroke survivors and the presence of deficits in ankle proprioception on the affected and unaffected sides in patients after stroke.

**Methods:**

Bilateral ankle proprioception was assessed in 20 stroke patients and 20 age-matched healthy controls using AMEDA. Test-retest reliability was assessed using the intraclass correlation coefficient (ICC).

**Results:**

The ICC in the affected and unaffected sides was 0.713 and 0.74, respectively. Analysis of variance revealed significant deficits in ankle proprioception in subacute stroke survivors vs. healthy controls (F = 2.719, *p* = 0.045). However, there were no significant differences in proprioception acuity scores between the affected and unaffected sides in patients after stroke (F = 1.14, *p* = 0.331).

**Conclusions:**

Stroke survivors had bilateral deficits in ankle proprioceptive acuity during active movements compared with age-matched healthy controls, underscoring the need to evaluate these deficits on both sides of the body and develop effective sensorimotor rehabilitation methods for this patient population. The AMEDA can reliably determine bilateral ankle proprioceptive acuity in stroke survivors.

## Introduction

Proprioception, which is the perception of body position and movement (1), is bilaterally reduced after unilateral stroke (2–5). In fact, to date, the majority of bilateral proprioceptive studies for stroke survivors focused on the upper limb (2–5), while fewer studies investigated proprioception in the lower limb (6, 7) and even fewer in the ankle (8, 9). Deficient suitable evaluation methods could be one reason for the lack of understanding characteristics of bilateral ankle proprioceptive impairment after stroke (10, 11). A recent study reported that deficits in ankle proprioceptive function were the strongest predictor of balance dysfunction after stroke (12), but different evaluation methods were reported to show different proprioceptive performance (13). Therefore, it is essential to find appropriate evaluation methods and understand the general characteristics of bilateral ankle proprioceptive function after stroke.

Clinical evaluation methods of ankle proprioception in stroke survivors are usually performed superficially (14, 15), including the Sensory subscale of the Fugl-Meyer-Scale (Fugl-Meyer), Revised version of the Nottingham Sensory Assessment (Revised NSA), Erasmus modifications to the revised Nottingham Sensory Assessment (Em-NSA), and Rivermead assessment of sensory performance (RASP). These classic scales are mainly for differential screening, which means it is only to assess whether there is a decline in proprioception, and it is not designed to quantify the severity of the decline. The threshold of detection of passive motion (TTDPM) (8) and joint position reproduction (JPR) (9, 16) are used to evaluate bilateral ankle proprioception for stroke survivors by using mechanical pieces of equipment [such as Biodex^TM^ Isokinetic Systems (9)]. However, during these studies, all the participants are required to wear eye masks, which is a highly artificial situation. It is argued that this kind of method isolates the proprioception from the version, which does not match the actual surroundings in daily life (17, 18). Besides, both of these methods present with relatively high equipment and time cost, and effort constraints limit terms of applicability in clinical and large population studies. Alternatively, active movement extent discrimination apparatus (AMEDA) is one of the typical proprioception assessment methods, which is economic and easy to use and has shown excellent ecological (17). It presents with sufficient sensitivity to evaluate ankle proprioceptive acuity in healthy elder adults (19) and healthy young adults (20), chronic ankle instability (21, 22), neurological degeneration associated with aging, and Parkinson's disease (23, 24). However, considering this is the first study to use AMEDA in stroke survivors, we will include an assessment of the test-retest reliability and detect the standard error of the mean (SEM) and minimal detectable change (MDC).

Accordingly, we hypothesize that AMEDA is a reliable tool for assessing ankle proprioceptive acuity in stroke survivors. We also hypothesize that bilateral ankle proprioception is lower in stroke patients than in age-matched controls. These findings will help establish clearly certain essential characteristics for patients after stroke.

## Methods

The local institutional review committee approved this study. This study was registered in the Chinese Clinical Trial Registry (http://www.chictr.org.cn) and obtained the following clinical trial number (ChiCTR2100054720).

### Participants

All participants were recruited from the local hospital and given written informed consent before data collection. Hobart et al (25) reported that 20 sample sizes for the reliability study were stable and we referred to the previous similar study's sample (21). So, the sample of this study was decided that 20 stroke survivors and 20 age-matched healthy controls would be necessary. The inclusion criteria for stroke survivors were (1) experienced their first unilateral hemisphere stroke, (2) ability to understand written and oral information (Mini-Mental State Examination score higher than 24), (3) modified Ashworth scale score lower than 2, (4) ability to understand and complete the AMEDA (have at least 16 degrees in ankle inversion). The exclusion criteria were cerebellar injury or vestibular disease. The age-matched healthy controls were recruited *via* local media advertisements, and ankle injuries in the past 6 months were the exclusion criteria. All participants needed to complete the Chinese version of the Edinburgh Handedness Inventory and Waterloo Footedness Questionnaire (Revised) (26).

**Figure 1:**
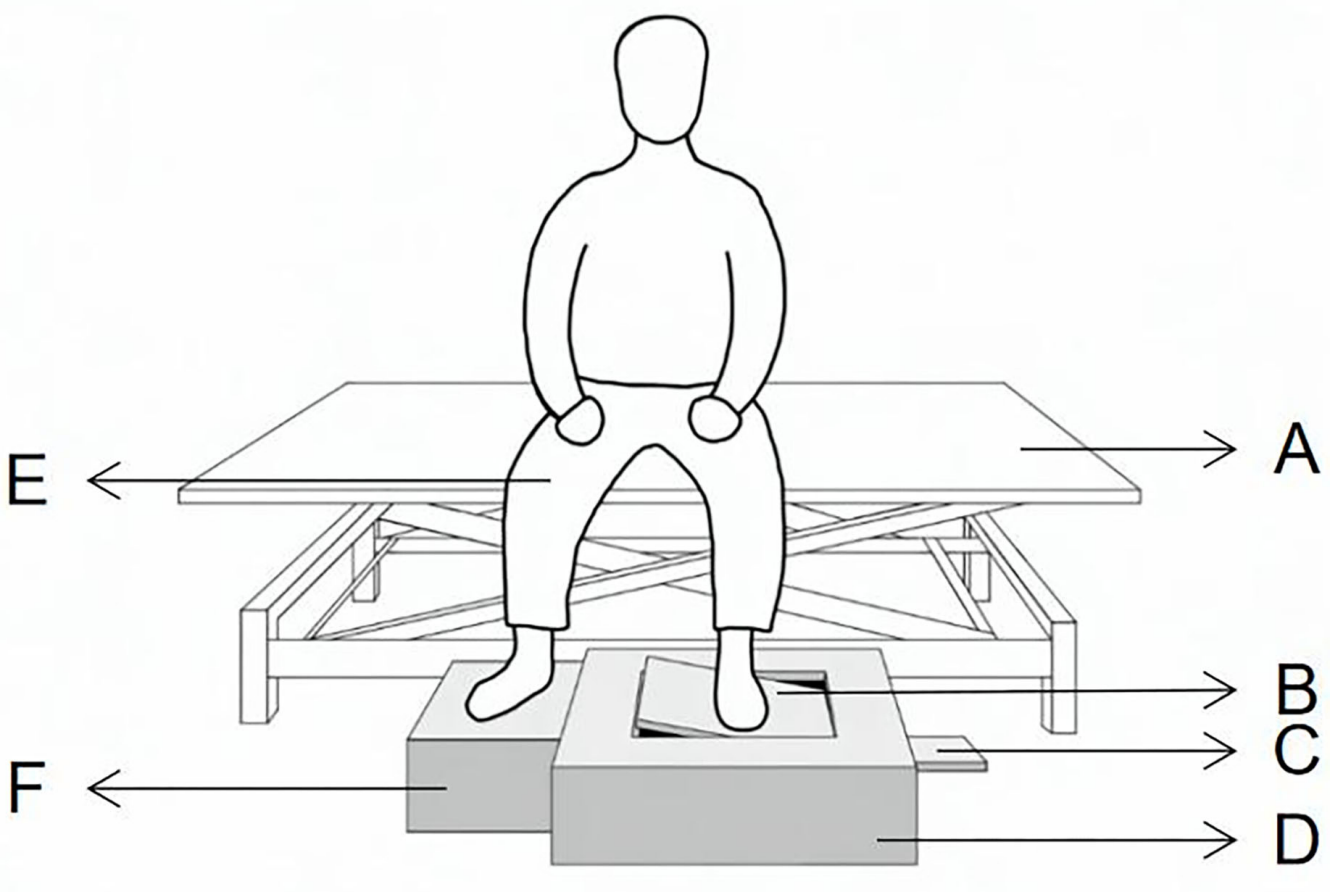
Active movement extent discrimination apparatus for assessing ankle proprioceptive acuity in the sitting position. **(A)** the adjustable Bed, **(B)** movable foot platform, **(C)** adjustable panel, **(D)** AMEDA main body, **(E)** knees flexed at 90°, **(F)** stationary foot platform.

Proprioceptive acuity in the ankle joint of the affected and unaffected sides was evaluated using the AMEDA. The AMEDA has two wooden platforms, one is the movable platform, and the other is the stationary platform. The movable platform can be tilted to four different inversion angles (10, 12, 14, and 16 degrees). From smallest to largest, each inversion angle is named as positions one, two, three, and four (Figure 1).

Since this is the first study to use AMEDA in Stroke Survivors, we did concern about its safety. So, to ensure safety in this study, the participants are asked to remain seated upright, with hips and knees flexed at 90°, and heads straight to avoid reliance on visual cues (27). During each trial, the participant was asked to use the test foot to actively tilt the movable platform until it reached one of the four predetermined angles and then return the platform to the original flat position, while the other foot remained on the stationary platform. One test included the familiarization period and the formal testing period. Each ankle inversion position was presented to the participant three times (12 trials) with the correct position number during the familiarization period and ten times in random order (40 trials) during the formal testing period. In the formal testing period, the participant was asked to subjectively answer the position number after each active inversion and eversion movement by recalling the positions from the familiarization trials (18). Both the affected side and the unaffected side ankle were tested with 1-min rest between, and the first test side was chosen at random. The 20 stroke survivors accepted the tests again 1 day later for the test-retest reliability.

### Data analysis

All data analyses used SPSS version 26 (IBM Corporation, Somers, NY), and all figures used GraphPad Prism version 8 (San Diego, CA). The area under the receiver operating characteristic curve (AUC) values were calculated to represent the proprioceptive acuity scores. A score of 1 indicated 100% accuracy, and 0.5 indicated that the accuracy was attributed to chance (28, 29). Significant differences between body sides and study groups were analyzed by one-way ANOVA. The level of significance was set at 5%. The normality of data distributions was assessed using the Shapiro test. Data were expressed as mean (standard error, SD) for normally-distributed continuous variables.

Test-retest reliability was calculated by intraclass correlation coefficient (ICC) (1, 2) (30, 31). ICC values of ≤0.49, 0.5–0.74, 0.75–0.9, and >0.9 indicated poor, moderate, good, and excellent reliability, respectively (30, 31). The standard error of the mean (SEM) was calculated to reflect within-subject variability and was obtained using the formula SEM = SD^*^√(1-ICC), where SD is √[SS_TOTAL_/(n-1)] (30, 32). The minimal detectable change at the 95% confidence level (MDC_95_) was calculated to evaluate the change scores in terms of variability of measure and was obtained using the formula: MDC_95_ = 1.96 × √2 × SEM, where 1.96 is the Z-score for 95% confidence interval (CI) (33, 34).

## Results

All the 40 participants completed all the tests and were all right-handed and right-footed and have Brunnstrom score higher than 3. The demographic characteristics of our cohort are shown in Table 1, including gender, age, days after stroke, lesion location (left hemiplegia or right hemiplegia), and post-stroke duration.

**Table 1:** Demographic characteristics of our cohort.

**Characteristic**	**Stroke patients**	**Healthy controls**	**Difference between groups**
Participants, *n*	20	20	/
Gender, *n*, male: female	17:3	17:3	Chi^2^ = 0, *p* = 1
Age, years, mean (SD)	61.20 (7.99)	60.85 (7.77)	t = 0.140, *p* = 0.889
Time after stroke, days (SD)	52.65 (36.85)	/	/
Leision location, left hemiplegia: right hemiplegia	10:10	/	/
Body mass index, mean (SD)	25.35 (2.07)	25.08 (3.29)	t = 0.318, *p* = 0.752

SD, standard error.

All data were normally distributed. The ICC was 0.713 (95% CI = 0.399–0.876, SEM = 0.054, MDC_95_ = 0.149) on the affected side and 0.74 (95% CI = 0.439–0.889, SEM = 0.041, MDC_95_ = 0.114) on the unaffected side.

The AUC values representing ankle proprioceptive acuity in stroke patients mean (SD) for the test and retest were 0.674 (0.1) and 0.673 (0.085) on the affected side and 0.676 (0.081) and 0.7 (0.072) on the unaffected side, respectively. The AUC values in healthy controls mean (SD) for the test were 0.747 (0.064) and 0.750 (0.067) on the non-dominant side and 0.738 (0.079) and 0.755 (0.057) on the dominant side, respectively.

**Figure 2:**
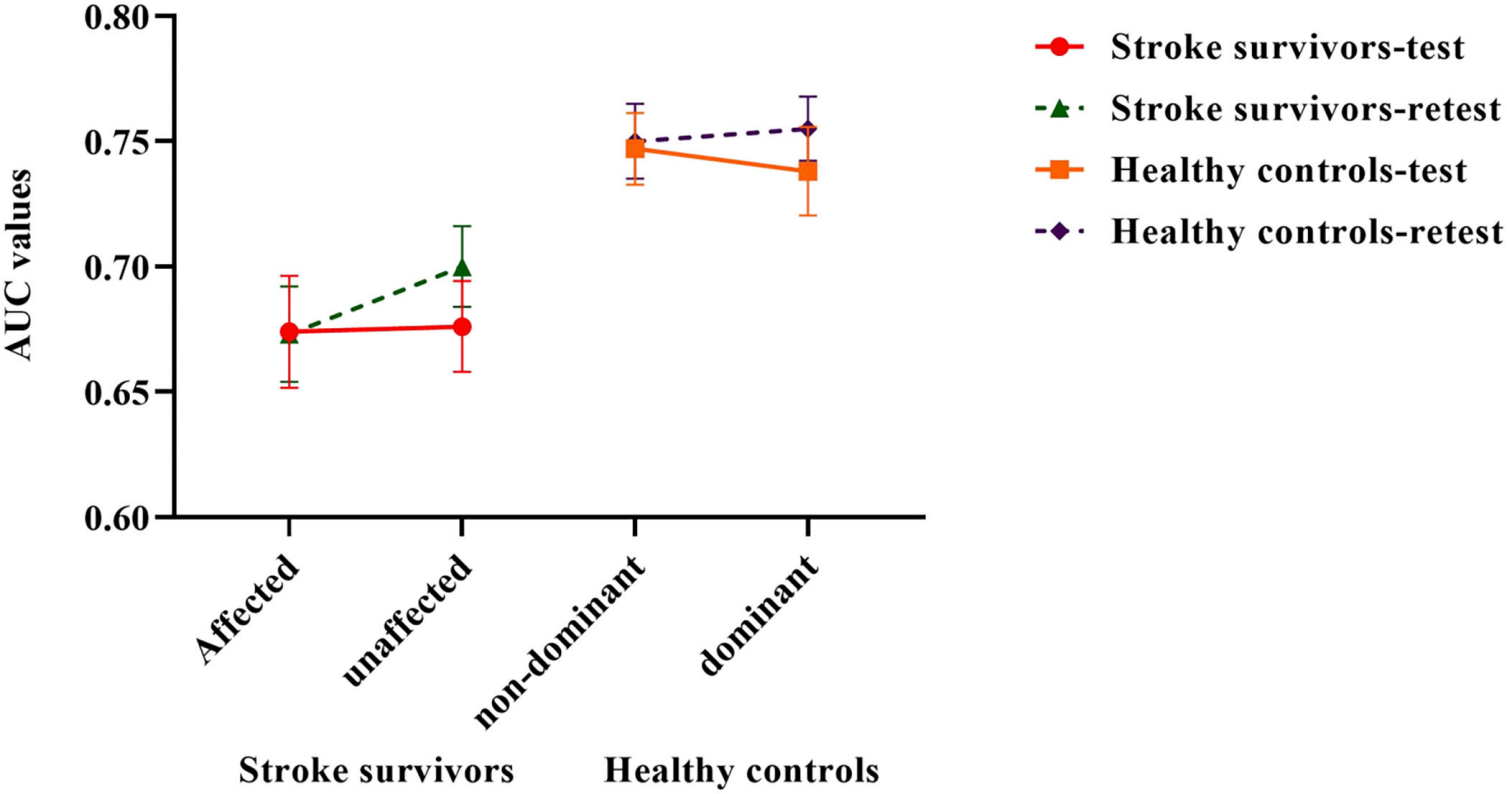
Ankle proprioceptive acuity scores in stroke survivors and healthy controls. Error bars represent standard errors of the mean.

There was no significant difference in proprioceptive acuity scores between the two sides (*p* > 0.05). The proprioceptive acuity scores were significantly lower in stroke survivors than in healthy controls (F = 2.719, *p* = 0.045, partial η^2^ = 0.237). ANOVA revealed that there was no significant difference in proprioceptive acuity scores between the affected and unaffected side of stroke survivors (F = 1.14, *p* = 0.331) (Figure 2).

## Discussion

In this study, ankle proprioceptive acuity on both the affected side and the unaffected side was significantly lower in stroke survivors than in healthy controls, demonstrating the presence of bilateral deficits in these stroke survivors. Our data does not agree with a previous study (8), which reported that the ankle proprioception acuity assessed by the TTDPM method on the unaffected side was not significantly different from the healthy controls. One reason may be due to the passive movement in TTDPM and active movement in AMEDA, suggesting that passive proprioception (less dependent on muscle contraction) is not affected by stroke (35). Another possible explanation may be the presence of separate processing of central neuro mechanisms for the two distinct submodalities of proprioception (36) (kinesthesia tested by TTDPM and position sense tested by AMEDA). A finding that also matched our previous study (13, 37) was that agreement between ankle proprioception scores of different methods in the same patient was weak, suggesting that different neural mechanisms underlie proprioceptive control. Different methods of proprioception should be viewed as complementary rather than interchangeable (38). Our findings indicate that bilateral active ankle position sense was deficient after unilateral stroke.

Moreover, the bilateral deficits in ankle proprioception are also in line with the neuroanatomy of the proprioception pathway (39). Bilateral premotor cortical and subcortical regions and contralateral sensorimotor cortex are involved in the sensory processing of proprioceptive input (5, 40, 41). The neurological system processes the sensory inputs and makes movement plans, and impulses are then conducted from the brain and spinal cord to muscles and glands, called motor output (42). In our study, AMEDA required active ankle movement output which could be influenced by proprioceptive inputs (20, 40, 43, 44). Also, it is in line with the theory of interhemispheric information transfer involving the corpus callosum, which means the proprioception information could be transferred between the left and right brain hemispheres, even after stroke (45–48).

In addition, Ankle proprioception is a significant determinant of balance because the foot and ankle joints are the main body parts in contact with the ground (49). Moreover, ankle proprioception deficit was reported as the strongest factor in the prediction of balance impairment in chronic stroke (12). Sensorimotor training improves lower limb proprioception and dynamic anterior-posterior balance in stroke patients (50). In this study, we did not do the predicted analysis and correlation study, but considering AMEDA was much more ecological (17), the ankle proprioception deficits measured by AMEDA were expected to have predicted balance impairment in subacute stroke patients. For this reason, we recommend further studies could work on it and assess the effect of bilateral training programs for ankle proprioception on motor functions in stroke survivors. Our data may provide a basis for developing effective bilateral treatments for proprioceptive and functional deficits.

To the best of our knowledge, this study is the first to use this apparatus to evaluate ankle proprioception in stroke survivors. The test-retest reliability of the AMEDA in our cohort was moderate (ICC > 0.5), demonstrating that this tool can reliably determine ankle proprioceptive acuity in stroke patients. In addition, this tool measures ankle proprioception in both the dorsal-plantar flexion and inversion-eversion plane (24). Thus, future studies should estimate this parameter in stroke patients in both planes to detect more characteristics of ankle proprioception. Besides, the lower limb spasticity after stroke is common and it can affect the balance, increase the risk of falling, and reduces the quality of life, we recommend further study to detect the effects of spasticity severity on the ankle in both dorsal-plantar flexion and inversion-eversion plane and its correlation with ankle proprioception and balance in the stroke survivors.

## Limitations

This study has several limitations: (1) cerebral hemorrhagic patients were not successfully enrolled due to the inpatients' unsuitable conditions during the study period, (2) stroke survivors were not classified according to the affected brain region due to the small sample size, and (3) only included the participants whose Ashworth score were lower than 2.

## Conclusion

Stroke survivors had impaired ankle proprioceptive acuity on the affected and unaffected sides compared with age-matched healthy controls. The AMEDA is a reliable tool for assessing ankle proprioceptive acuity in stroke survivors.

## Data availability statement

The raw data supporting the conclusions of this article will be made available by the authors, without undue reservation.

## Ethics statement

The studies involving human participants were reviewed and approved by the Research Ethics Committee of Huashan Hospital Affiliated to Fudan University (Shanghai, China). The patients/participants provided their written informed consent to participate in this study.

## Author contributions

LP designed the study, collected data, analyzed data, and drafted the manuscript. YZ and JH participated in the study design, data analysis, and helped to draft the manuscript. DX, WW, and JR helped with study design, data collection, and data analysis. JX, AF, and RA made edits and comments to the manuscript. All authors conceived of the study, read and approved the final version of the manuscript, agreed with the order, and presentation of the authors.

## Conflict of interest

The authors declare that the research was conducted in the absence of any commercial or financial relationships that could be construed as a potential conflict of interest.

## Publisher's note

All claims expressed in this article are solely those of the authors and do not necessarily represent those of their affiliated organizations, or those of the publisher, the editors and the reviewers. Any product that may be evaluated in this article, or claim that may be made by its manufacturer, is not guaranteed or endorsed by the publisher.
